# ﻿A new species of *Atriplex* (Amaranthaceae) from the Indian subcontinent

**DOI:** 10.3897/phytokeys.229.105162

**Published:** 2023-07-27

**Authors:** Alexander P. Sukhorukov, Nidhan Singh, Maria Kushunina, Maxim A. Zaika, Alexander N. Sennikov

**Affiliations:** 1 Department of Higher Plants, Biological Faculty, Moscow State University, Leninskie Gory 1/12, Moscow, 119234, Russia Moscow State University Moscow Russia; 2 Laboratory Herbarium (TK), Tomsk State University, Lenin Ave. 36, Tomsk, 634050, Russia Tomsk State University Tomsk Russia; 3 Department of Botany, I.B. College, Panipat, Haryana-136021, India I.B. College Panipat India; 4 Department of Plant Physiology, Biological Faculty, Lomonosov Moscow State University, Moscow, 119234, Russia Lomonosov Moscow State University Moscow Russia; 5 Botanical Museum, Finnish Museum of Natural History, University of Helsinki, 00014, Helsinki, Finland University of Helsinki Helsinki Finland

**Keywords:** *Atriplex*, Chenopodiaceae-Amaranthaceae, Indian subcontinent, new species

## Abstract

A new subshrubby C_4_-species from the lowlands and foothills of India, Pakistan and SE Afghanistan, *Atriplexpseudotatarica*, is described and illustrated. Previously, it was incorrectly identified as *A.crassifolia* auct. non C.A.Mey. belonging to a distant C_3_-group of the genus. A phylogenetic analysis based on nrITS and nrETS revealed its position as sister to *A.schugnanica* (sect. Obionopsis). Both species share aphyllous inflorescence and smooth bract-like cover, but differ in life form, leaves, seed colour, and geographical distribution. We revised native Indian *Atriplex* species and excluded some of them from the flora of the country. An improved checklist of the native *Atriplex* species in India with their corrected synonymy and nomenclature is given, and a new diagnostic key is provided.

## ﻿Introduction

*Atriplex* L. is the largest genus in the Amaranthaceae clade encompassing ca. 260 species distributed mostly in arid regions of the world (Žerdoner Čalasan et al. 2022). There is a relatively limited number of *Atriplex* species in the Indian subcontinent. The latest treatments for the flora of Pakistan (Ali and Qaiser 2001) and India (Paul 2012) counted 12 and seven species, respectively. Sukhorukov et al. (2019) revised *Atriplex* in the Himalayas and Tibet, and provided many taxonomic changes for the genus in the Indian Himalaya compared to the previous checklists and floras. All *Atriplex* species native to the Himalayas are represented by the annual C_4_ species, but they have different origins. Two of them, *A.pamirica* Iljin and *A.centralasiatica* Iljin, are typical Central Asian elements, whereas *A.schugnanica* Iljin originated in the eastern Irano-Turanian region (Žerdoner Čalasan et al. 2022). Subsequently, the first two species are classified within A.sect.Obione (Gaertn.) Reichenb., and *A.schugnanica* is a member of A.sect.Obionopsis (Lange) Dumort. (Sukhorukov et al. 2022). In comparison to the species distributed in the Himalayas, the species growing in lowlands and foothills of the Indian subcontinent are still undercollected and poorly known, because the classical authors preferred to stay in the mountains rather than in the plains during the summer time due to harsh climatic conditions in the latter region, and had a preference for species-rich plant diversity in the mountains.

Unusual *Atriplex* plants were noted in the year 2021 in Haryana State (India) by one of the authors (NS) of the present paper. Further *in situ* studies have confirmed an assumption that the specimens cannot be assigned to any known species or their synonyms, and should be described as a new species.

## ﻿Materials and methods

### ﻿Material investigated

Field studies were carried out in the Haryana State (India). Taxonomic revision of the herbarium material was undertaken in the herbaria BM, CAL (examined as digital images), DD, K, LE, MHA, and MW. Distribution map is based on the specimens cited in the text and was prepared using SimpleMappr online tool (http://www.simplemappr.net).

### ﻿Sampling of the study, DNA extractions, amplification, and sequencing

Thirty-seven accession numbers were included in the phylogenetic analyses representing *Atriplex* species, and two accession numbers were taken as outgroups from Amaranthaceae. The samples are listed in Table 1. We have reconstructed a part of the global phylogenetic tree originally published by Žerdoner Čalasan et al. (2022) and indicated the position of the new species among its close relatives.

**Table 1.** GenBank accession numbers for the species of *Atriplex* and an outgroup included in the phylogenetic analysis.

Species	ITS	ETS
*A.dimorphostegia 377*	OM180193	OM179544
*A.flabellum 4591*	OM180202	OM179553
*A.fominii 4216*	OM180203	OM179554
*A.kalafganica 4223*	–	OM179575
*A.laciniata 4357*	OM180227	OM179577
*A.lasiantha 4221*	OM180231	OM179580
*A.moneta 4592*	OM180253	OM179599
*A.olivieri 4229*	OM180268	OM179612
*A.ornata 4508*	OM180270	OM179614
*A.paradoxa 3917*	OM180276	OM179620
***A.pseudotatarica*** *9*	OQ843457	OQ829353
*A.pratovii 4236*	OM180288	OM179631
*A.pungens 4365*	OM180292	OM179635
*A.recurva EM391*	OM180298	OM179641
*A.schugnanica 4367*	OM180307	OM179648
*A.tatarica 4570*	OM180325	OM179665
*A.tataricavar.pseudoornata 4373*	OM180326	OM179666
*A.tornabenei 4375*	OM180327	OM179667
Outgroup		
*Halimionepedunculata* s.n.	OM180349	OM179688

The number next to the taxon indicates the voucher (see Žerdoner Čalasan et al. 2022). We highlighted in bold the binomial of the new species.

Among 16 species analyzed in A.sect.Obionopsis and close relatives (*A.flabellum* Bunge, *A.moneta* Bunge), 15 accessions were represented by ITS and ETS loci (see below) (Table 1). We included only ETS sequences for one species (*A.kalafganica*). Following Kadereit et al. (2010), we selected *Halimionepedunculata* (L.) Aellen as an outgroup for ITS- and ETS-based molecular phylogenetic analyses. In short, we analyzed 37 ITS and ETS sequences of 19 taxa (Table 1). We obtained two of these sequences (one of ITS and one of the ETS regions of rDNA) as a part of this study (Table 1) and took the remaining ones from the study of Žerdoner Čalasan et al. (2022).

The DNA from a sample of *A.pseudotatarica* collected in the state of Haryana, India (see also the Results section) was extracted from 5–10 mg of dried leaves employing the DNeasy Plant Mini Kit (Qiagen, the city of Valencia, CA, USA), as described in the manual.

PCRs were carried out in Thermal Cycler T100 (Bio-Rad, USA) using the primers and cycling protocols summarized in Table 2.

**Table 2.** Primers and cycling protocols.

Marker	Primer	The source of primer	Cycling protocols (modified from Zacharias and Baldwin (2010))
ITS	Forward (ITS-5): 5’-GGA AGT AAA AGT CGT AAC AAG G-3’	White et al. (1990)	96 °C for 1 min; 40 cycles of (96 °C for 10 sec, 48 °C for 30 sec, and 72 °C for 20 sec + 4 sec/cycle); 72 °C for 5 min.
	Reverse (ITS-4): 5’-TCC TCC GCT TAT TGA TAT GC-3’		
ETS	Forward: (ETS-Atr): 5′-CAC GTG TGA GTG GTG ATT GGT T-3′	Zacharias and Baldwin (2010)	96 °C for 1 min; 40 cycles of (96 °C for 10 sec, 60 °C for 30 sec, and 72 °C for 20 sec + 4 sec/cycle); 72 °C for 5 min
	Reverse (18S-E): 5′-GCA GGA TCA ACC AGG TAG CA-3′	Baldwin and Markos (1998)	

The PCR cocktail (20 μL) contained 1.5–2 ng of the total DNA, 5 pmol of each primer, 4 μL of Ready-to-Use PCR Master mix 5× MasDDTaqMIX-2025 containing a “hot-start” SmarTaq DNA polymerase (Dialat Ltd., Moscow, Russiа).

PCR products were purified with the Cleanup Mini BC023S Kit (Evrogen, Russia) following the manufactured instructions. Sanger sequencing was performed at Evrogen JSC (Moscow, Russia) employing PCR primers (Table 2).

### ﻿Alignment and phylogenetic analyses

The L-INS-i alignment strategy with default settings of MAFFT version 7.0 (Katoh et al. 2017) was used to align sequences from both datasets (ITS and ETS). Two obtained alignments were manually edited and concatenated in program PhyDe version 0.9971 (Müller et al. 2010). The combined dataset (ITS and ETS) comprises 1032 bp (593 in ITS and 439 in ETS alignment) and 19 taxa.

We reconstructed the ITS plus ETS phylogeny of Atriplexsect.Obionopsis and two close relatives (*A.flabellum*, *A.moneta*) using the Maximum Likelihood approach (ML; Felsenstein 1973, 1983) and Bayesian Inference (BI; Rannala and Yang 1996). Gaps were treated as missing data. A variant of the General Time Reversal nucleotide substitution model (Tavaré 1986) (GTR + G+ I) was automatically selected by jModelTest v.2.0 (Darriba et al. 2012) for each partition (ITS and ETS) following the Akaike Information Criterion (AIC; Akaike 1974). For the ML analyses of concatenated alignment, we employed RAxML v.8 (Stamatakis 2014). ML Bootstrap analysis was conducted with 2500 replicates by the same program.

BI was performed in BEAST v.2.6.7 (Drummond et al. 2002; Bouckaert et al. 2014). Two runs with four chains each were run for 20 million generations for the combined dataset; both chains were sampled every 20.000 generations with a default parameter. Output log files were analysed using TRACER v.1.6 (Rambaut et al. 2014) to assess all parameters’ convergence and effective sample size (ESS). Ten percent of the samples were removed as burn-in. A maximum clade credibility tree was generated using TREE ANNOTATOR v.2.4.5 (Drummond and Rambaut 2007).

## ﻿Results

### ﻿ Atriplex pseudotatarica

Taxon classificationPlantaeCaryophyllalesAmaranthaceae

Sukhor. & Nidhan Singh sp. nov.

E1C11095-9CB0-5522-8F56-B9FD1D97F44B

urn:lsid:ipni.org:names:77324170-1

Fig. 1

– Atriplexcrassifolia auct. non C.A.Mey.
– Atriplexleucoclada auct. non Boiss.
– Atriplex spp. div. in herb. DD and K.

#### Type.

India. Haryana, surroundings of Panipat town, near Asan Kalan village, 29°15.1286'N, 76°31.4816'E, 15 Nov 2022 [in flowering and early fruiting stages], *N. Singh & A. Sukhorukov 9* (Holotype: CAL, isotype: BSD).

#### Description.

Monoecious subshrub up to 1.5 m high, branched in upper half; leaves alternate, shortly petiolate; petioles up to 1.0 cm long; blades greyish-silvery on both sides, 1.0–4.0 × 0.5–1.0 cm (much smaller towards inflorescence), oblong or narrowly oblong, entire or shallowly sinuate, with Kranz-anatomy; inflorescences branched, up to 15 cm long, with pseudopposite bracts or with a few small leaves forming pseudowhorls (after fruiting turning into small alternate leaves in younger shoots), aphyllous in other parts; glomerules condensed or slightly interrupted, of both male and female flowers, the latter are also located at the axils of uppermost leaves below the main inflorescence; male flowers stipitate at base, with 5 free perianth segments, anthers 0.25 mm long; bract-like cover of female flowers (Fig. 2A, B) rhombic, entire or scarcely dentate, with or without lateral angles, smooth at the back or rarely with 1–2 very short outgrowths, valves connate to the halfway, sometimes to one third of their length, with indistinct veins, heteromorphic in some other characters: (1) bract-like cover of female flowers located in leaf axils ± indurated in lower half and inflated at fruiting, 4.5–5.5 mm long, rhombic, and (2) bract-like cover of female flowers located in the main inflorescence slightly indurated and not inflated at fruiting, 2.0–4.0 mm long, trilobate and rhombic; seeds heterospermic (Fig. 2C, D): seeds developing in fruits located below the main inflorescence black, slightly elongated (1.1–1.3 × 1.4–1.6 mm), ripening earlier (in November; obs. in Haryana State, India) compared with those of the fruits located in the main inflorescence; seeds in fruits located in the main inflorescence black (similar to those developing below the main inflorescence) or yellowish-brown, 0.8–1.0 mm in diameter, ripening in late November–December.

**Figure 1.**
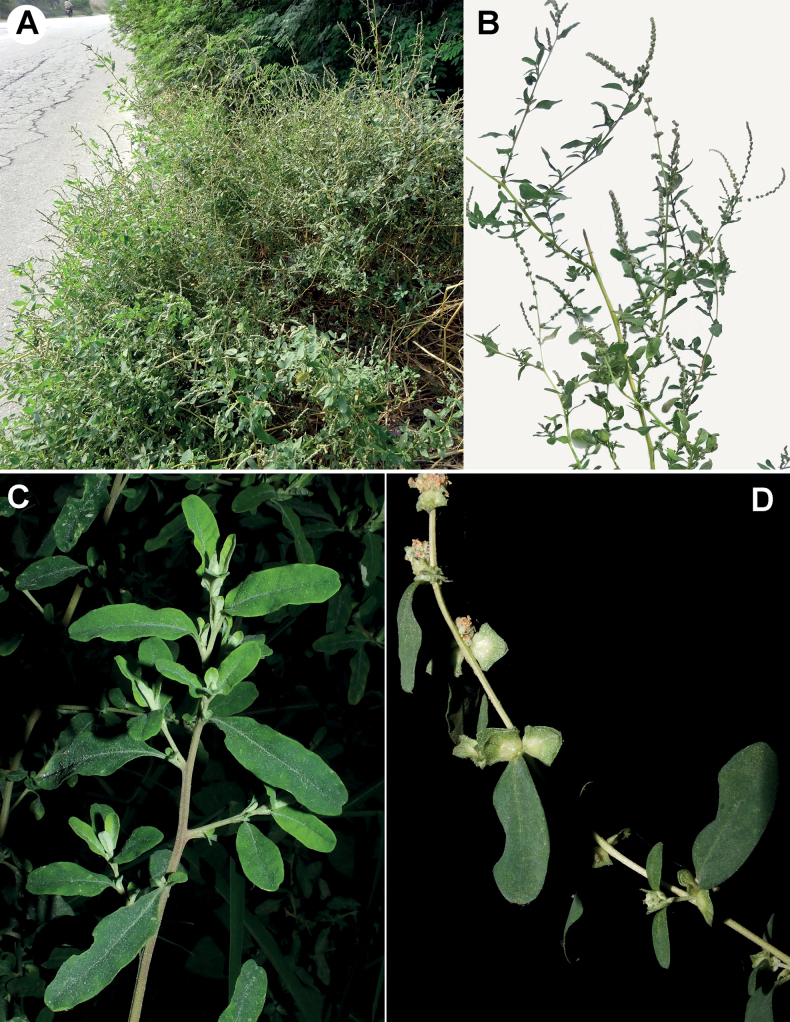
*Atriplexpseudotatarica*. **A** an overview of the plant **B** a twig with the inflorescence **C** a vegetative shoot **D** a shoot at fruiting. Origin of the material **A** Haryana, near Asan Khurd village, Nov 2022 **B** Haryana, near Asan Kalan village, Nov 2022 **C** Haryana, near Asan Kalan village, Aug 2022 **D** Haryana, near Panipat town, Oct 2014. Photographer: **A, B** A. Sukhorukov, **C, D** N. Singh.

**Figure 2.**
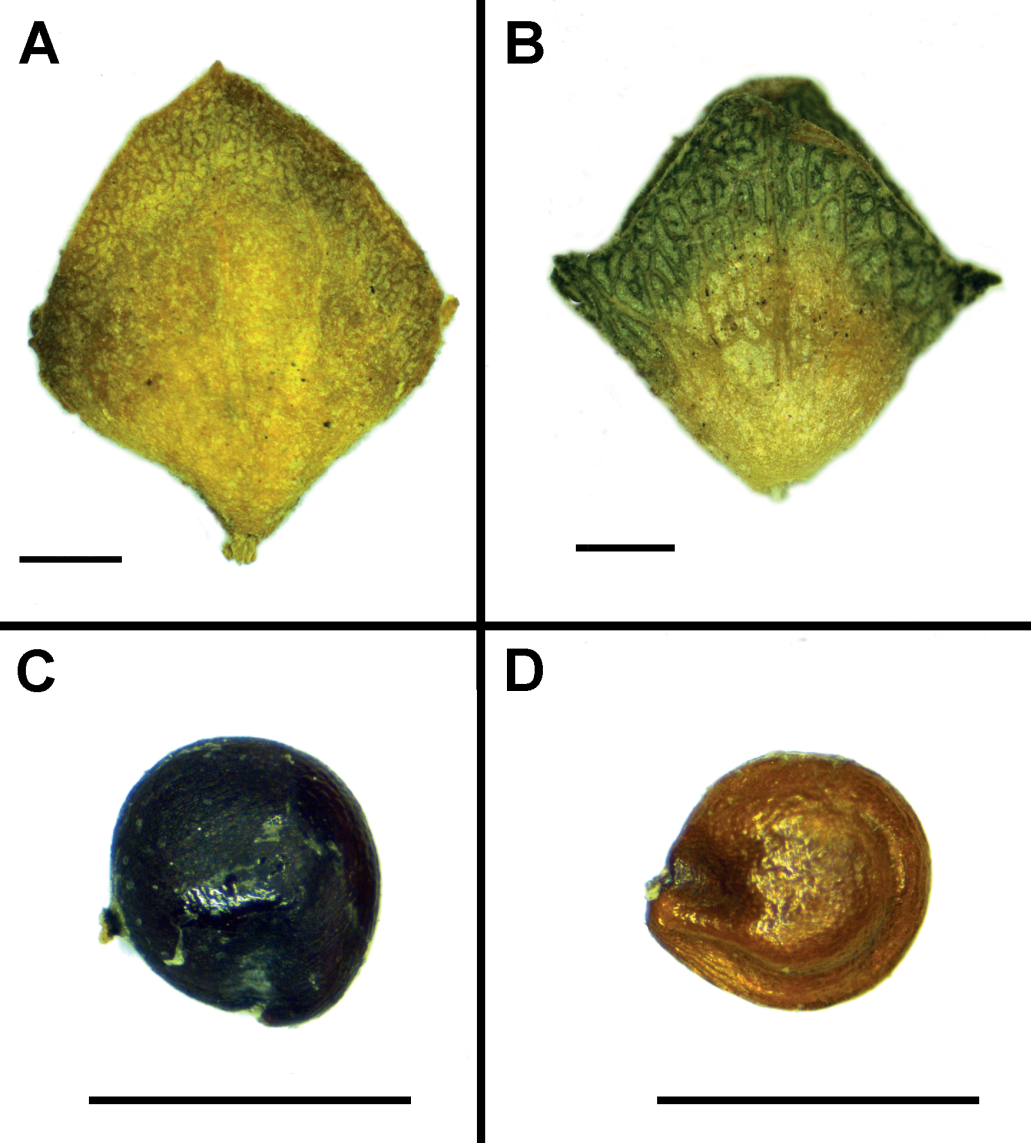
Bract-like cover (**A, B**) and seeds (**C, D**) of *A.pseudotatarica***A** cover of a female flower located below the main inflorescence **B** cover of a female flower located in the main inflorescence **C** black seed **D** yellowish-brown seed. Scale bars: 1 mm.

#### Phenology.

Flowering: July–November; fruiting: November–December.

#### Habitat.

Saline soils, sands, wasteland, roadsides, 0–2200 m a.s.l. In the natural landscapes in Haryana, *Atriplexpseudotatarica* was observed together with *Bassiaindica* (Wight) A.J.Scott, *Suaedafruticosa* Forssk. (all – Amaranthaceae), and some grasses.

#### Etymology.

The specific epithet is chosen due to the resemblance of the new species to *A.tatarica* L., which also has long aphyllous inflorescences.

#### Conservation status.

Although there is currently a limited number of collected specimens of *Atriplexpseudotatarica*, this species is clearly more overlooked than rare. Given that it is often found in disturbed habitats, produces a large number of seeds and is naturally adapted to saline substrates, we propose that the species should be assigned to the IUCN Red List category “Least Concern” (IUCN Standards and Petitions Committee 2022).

#### Distribution

**Figure 3.**
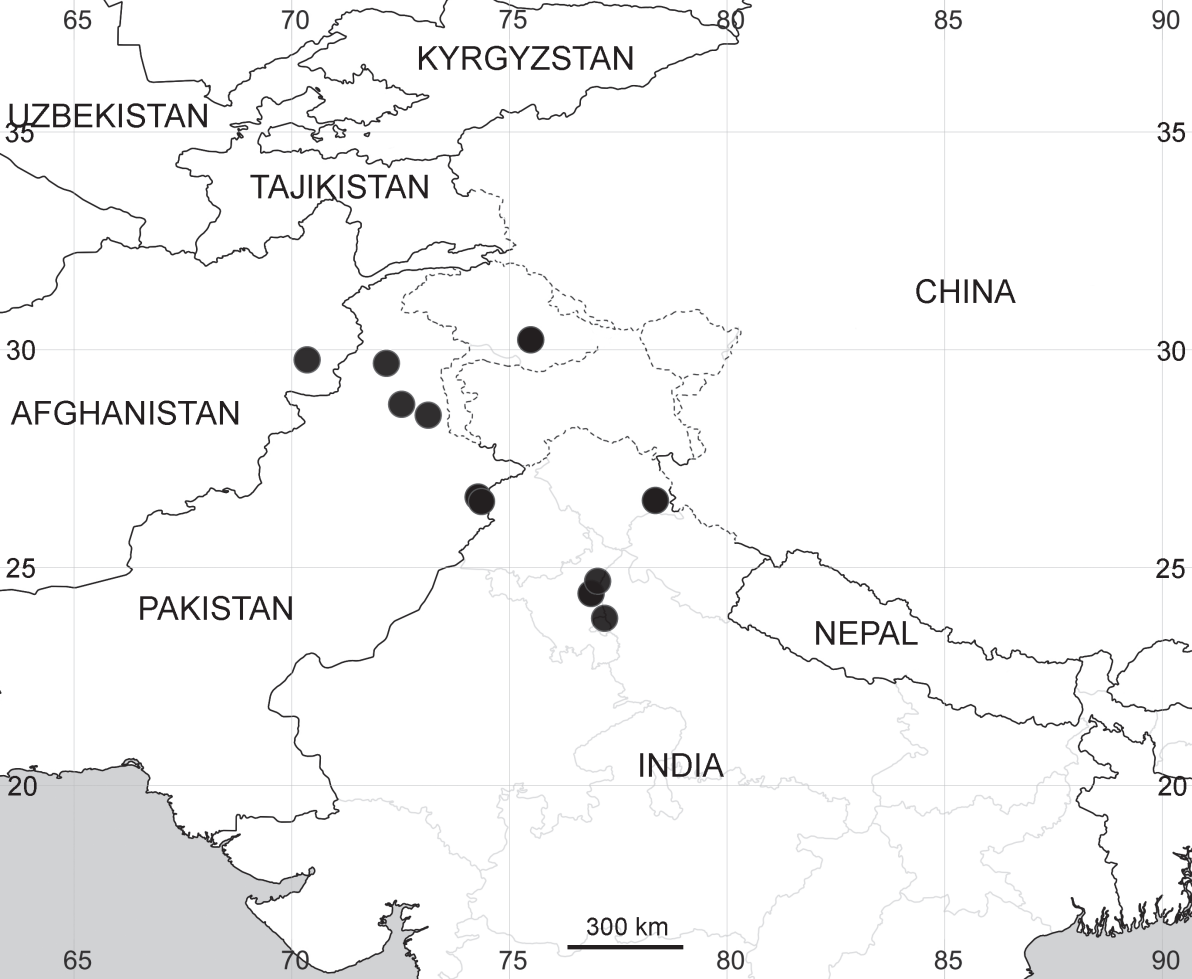
Records of *A.pseudotatarica*.

**(Fig. 3).** India, Pakistan and SE Afghanistan.

#### Specimens examined.

Afghanistan. [Laghman province] Alingar valley, 6000 ft, 1 Sep 1956, *W. Thesiger 1693* (BM).

India. Delhi, 23 Oct 1874, *anonymous 23395* (K); [Himachal Pradesh] Kimawar [Kinnaur], 1884, *J.R. Drummond 535* (DD-29978); [Haryana], Karnal, 1885, *J.R. Drummond 26479* (K); Haryana, surroundings of Panipat town, nr Asan Khurd village, 29°18.1584'N, 76°31.8779'E, 15 Nov 2022, *N. Singh & A. Sukhorukov 7* (CAL, BSD).

Pakistan. Lahore, 1846, *T.T. Thomson s.n*. (K); [Punjab] Rawalpindi, 1872, *J.E.T. Aitchinson 224* (K); [Gilgit-Baltistan prov.] Skardu, 7000–8000 ft, 15 Jul [18]92 [early flowering], *without collector’s name 12060* (DD!); [Khyber Pakhtunkhwa prov., Swat Distr.] Shohdara, 11 Nov 1935 [in fruiting stage], *R.R. Stewart 15362* (DD-77925); Lahore, common in weedy places, 17 Oct 1938 [in flowering stage], *Parker s3436* (DD-81928, DD-81929, K); [Punjab province], nr Attock, 15 Nov 1956, *R.R. Stewart 27830* (K).

#### Notes.

All examined herbarium specimens of *A.pseudotatarica* are represented by upper twigs of the plants. They were mostly incorrectly identified as *A.crassifolia*, or rarely left without identification, as *Atriplex* sp. To date, the name *A.crassifolia* may be found misapplied to some other species attributable to different groups of the genus. *Atriplexcrassifolia* is an annual C_3_-species belonging to A.sect.Teutliopsis Dumort. (Moser 1934; Iljin 1936; Sukhorukov 2006; Žerdoner Čalasan et al. 2022) with a restricted distribution range in semideserts of Kazakhstan and South-West Siberia, Russia (Iljin 1936; Sukhorukov 2006), penetrating into western China (Sukhorukov in Nobis et al. 2016). All other records of *A.crassifolia* reported from Europe are erroneous (Sukhorukov 2006; Sukhorukov et al. 2019). Aellen (1939), Ivanov (1989) and Medvedeva (1996) erroneously applied this name to *A.patens* (Litv.) Iljin, another species from A.sect.Teutliopsis (Sukhorukov 2006). The specimens from the Mediterranean area (GBIF Sekretariat 2022; re-identifications in BM!, K!, LE!) belong to *A.tornabenei* Tineo (C_4_-clade, A.sect.Obionopsis (Lange) Dumort.: Sukhorukov et al. 2022). The name *A.crassifolia* has also been widely used for the plants growing in lowlands of India and Pakistan (e.g., Hooker (1890), Bamber (1916)), and it is still erroneously applied in recent floras, checklists and ecological studies (Puri et al. 1964; Shetty and Singh 1991; Hussain and Mirza 1993; Jain et al. 2000; Kumar 2001; Paul 2012; Kumar and Singh 2013; Ibrahim 2019). Hooker (1890) stated that *A.crassifolia* is present in both lowlands (“westwards of Jumna [Yamuna] River”) and high mountains. Nevertheless, all records of *A.crassifolia* from the Himalayas refer to C_4_-species from A.sect.Obione (Gaertn.) Reichenb.: *A.pamirica* Iljin and *A.schugnanica* Iljin [= *A.pallida* (Moq.) Sukhor. ≡ *Chenopodiumpallidum* Moq., nom. rejic. prop.], and those from the lowlands and foothills belong to *A.pseudotatarica*.

Some of the plants growing in the lowlands of Pakistan also belong to *A.pseudotatarica*, of which some specimens were misidentified as *A.leucoclada* Boiss. Hedge (1997) noted that this species is highly polymorphic in the area covered in “Flora Iranica”, with the extreme forms having smooth, apically trilobate bract-like cover. Unfortunately, he did not indicate where such specimens were collected, but such characters are typical of *A.pseudotatarica*.

#### Phylogenetic relationships

**Figure 4.**
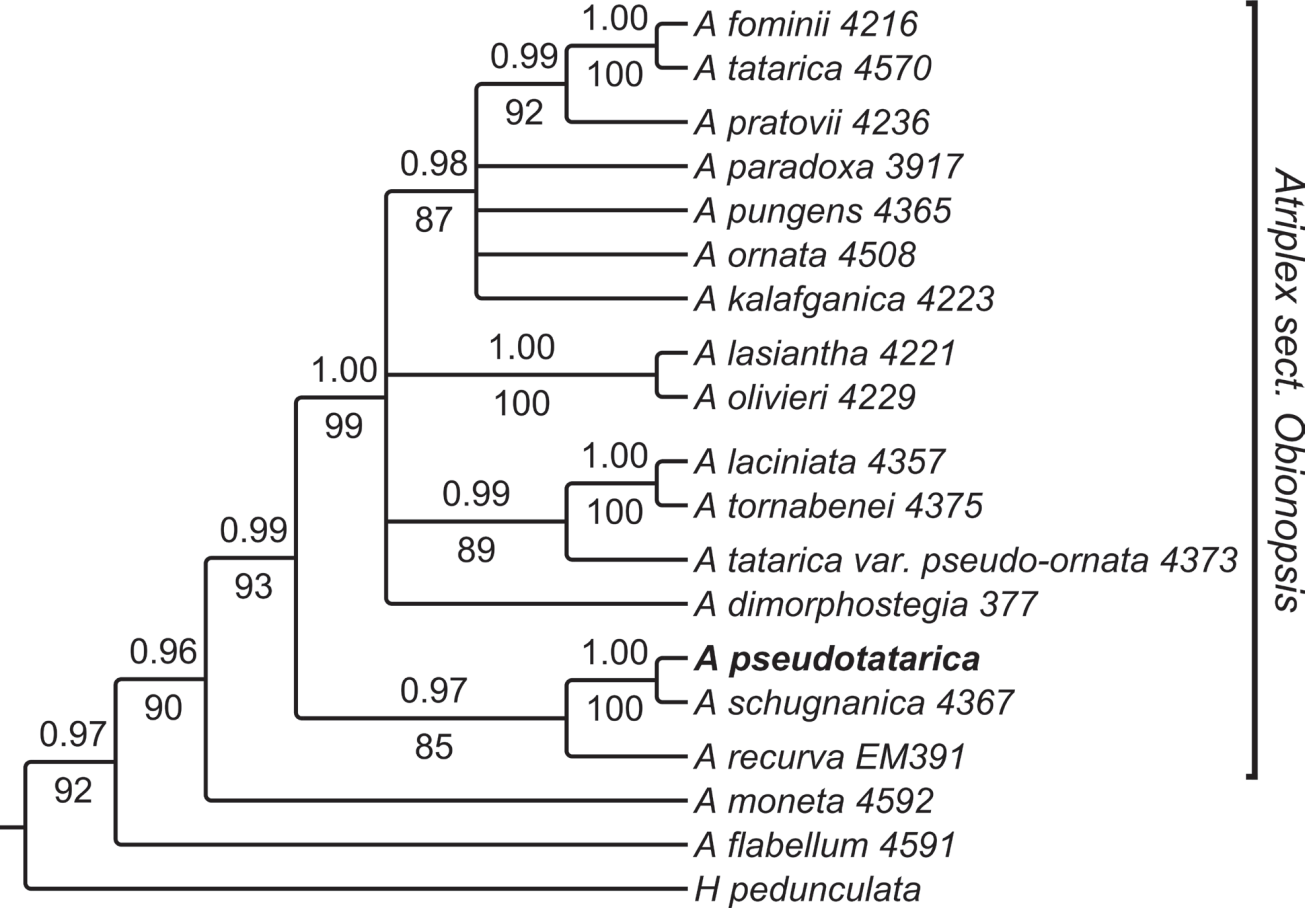
Maximum clade credibility tree from the BEAST analysis of the ITS+ETS *Atriplex* dataset. Bayesian posterior probabilities are given above the branches, bootstrap percentages of the maximum likelihood analyses are given below the branches.

**(Fig. 4).** Based on the combined nrITS and nrETS analyses, *A.pseudotatarica* was found sister to *A.schugnanica*, and both form a subclade within the Eurasian clade, A.sect.Obionopsis (Lange) Dumort., which comprises ~ 15 C_4_-species predominantly distributed in Irano-Turanian floristic region (Sukhorukov et al. 2022; Žerdoner Čalasan et al. 2022). *Atriplexpseudotatarica* and *A.schugnanica* share some characters typical of the members of A.sect.Obionopsis (aphyllous inflorescences, sclerified bract-like cover with the valves connate up to the half of their length, presence of the female flowers in leaf axils and both female and male flowers in the inflorescence), but have several conspicuous morphological differences (Table 3). Additionally, *A.pseudotatarica* is distributed in the lowlands and pre-Himalayan foothills, whereas *A.schugnanica* is a typical montane plant growing in the West Himalayas, Karakoram and Pamir at the altitudes of (2000) 2600–4800 m a.s.l. (Iljin 1936; Sukhorukov et al. 2019). In Table 3, we also included other similarly looking C_4_*Atriplex* species; three of them (except *A.tatarica*) are present in the lowlands of Pakistan, and only one (*A.pseudotatarica*) is reaching India. *Atriplextatarica*, widely distributed in many parts of the “Flora Iranica” area, as well as *A.kalafganica* Aellen (Aellen in Podlech 1975) are also added here because of their morphological resemblance with *A.pseudotatarica*.

**Table 3.** The diagnostic features of *A.pseudotatarica* and similarly looking C_4_*Atriplex* species.

Species / Character	Life form	Leaves	Bract-like cover	Seeds
*A.kalafganica*	Annual	shortly petiolate, rhombic or ovate, entire or dentate; upper leaves do not form pseudowhorls	with small or prominent outgrowths or smooth	red and brown
*A.lasiantha*	Annual	petiolate, rhombic or ovate, entire or dentate; upper leaves do not form pseudowhorls	with outgrowths or smooth	red and brown
*A.leucoclada*	Subshrub	sessile (except lowermost leaves), triangular-deltoid, situate-dentate; upper leaves do not form pseudowhorls	with outgrowths	dark brown
*A.pseudotatarica*	Subshrub	shortly petiolate, oblong, (sub)entire; upper leaves pseudopposite or forming pseudowhorls at fruiting	smooth or with 1–2 small outgrowths	black and brown
*A.schugnanica*	Annual	petiolate, triangular or rhombic; upper leaves do not form pseudowhorls	smooth or with 1–2 small outgrowths	red and brown
*A.tatarica*	Annual	petiolate, rhombic, triangular, rarely lanceolate, entire to erose-dentate or lobate; upper leaves do not form pseudowhorls	with small or prominent outgrowths or smooth	red and brown

The most conspicuous characters of *A.pseudotatarica* are subshrubby life form, pseudopposite leaves or leaves in pseudowhorls below the inflorescence seen at fruiting, and presence of black seeds.

## ﻿Discussion

### ﻿Genus *Atriplex* in India

A recent revision of the genus in the Himalayan area (Jammu and Kashmir, Himachal Pradesh, Uttarakhand States) has been provided by Sukhorukov et al. (2019, with references therein), and some species (*A.crassifolia*, *A.laciniata* L., *A.rosea* L., *A.sagittata* Borkh. [previously known as *A.nitens* Schkuhr: Paul (2012)] were excluded from this area. The Chenopodiaceae of lowland India are still poorly studied and have not been included in any detailed morphological and chorological studies. Additionally, the plant material from India is old and quite scarce in the European herbaria. All these factors influenced a poor knowledge of some genera like *Atriplex*. Below we provide an improved taxonomic survey of *Atriplex* in the tropical part of India, with some notes on alien species of the genus.

Several alien species of Australian and North American origin were reported from India: *A.amnicola* Paul G.Wilson, *A.nummularia* Lindl., *A.lentiformis* (Torr.) S.Watson (Rani et al. 2013; Kumar et al. 2021). As stated by Singh (2005), many areas in India, especially influenced by a monsoon, are unfavourable for (semi)desert plants such as *Atriplex*. The Rajasthan State and some adjacent areas are of particular interest because they lie in the desert zone that is suitable for *Atriplex* species. We did not find any *Atriplex* specimens in Rajasthan, but several species were reported from this region including subshrubby American *A.lentiformis* (Gupta and Arya 1995), European *A.hortensis* (Bole and Pathak 1988) and two native species, subshrubby *A.stocksii* (Wight) Boiss. (Puri et al. 1964; Bole and Pathak 1988; Arya and Lohara 2016) and annual *A.* “*crassifolia*” (Puri et al. 1964; Shetty and Singh 1991). *Atriplexcrassifolia* sensu Puri et al. (1964) was reported from the vicinity of Jodhpur and described in the diagnostic key as “an annual, male flower clusters axillary or in short leafy spikes”, but elsewhere these authors provided a different diagnosis (“male flower clusters in slender leafless interrupted spikes”). We were unable to trace which species should be recognised instead of *A.crassifolia* because these contradictory diagnoses cannot be applied to any species. Shetty and Singh (1991) described it as an annual species with interrupted inflorescences and inflated bract-like covers. These two reproductive characters are also found in *A.pseudotatarica*, but the life form is different in the latter species. Nevertheless, we presume that *A.pseudotatarica* may be present in both Rajasthan and Gujarat due to the records in Haryana State.

### ﻿Key to *Atriplex* species native to India

All native species have the C_4_ leaf anatomy. No C_3_*Atriplex* species were recorded in India. The alien species are not included in the key because their taxonomy and alien status have not been fully evaluated.

**Table:** 

1	Stems procumbent, rooting at nodes; leaves (sub)opposite, at least in upper part of the branches, entire (species growing in southern India)	***A.repens***
–	Stems erect, not rooting at nodes; leaves alternate, usually undulate, dentate or lobate (species from western, central and northern parts of India)	**2**
2	Valves of bract-like cover almost free, orbicular; coastal subshrubby plants from western India	***A.stocksii***
–	Valves rhombic, connate to the half of their length	**3**
3	Inflorescence aphyllous or bracteate	**4**
–	Inflorescence leafy (almost) to the top	**5**
4	Annual growing at high altitudes (2600–4800 m a.s.l.); leaves triangular or oblong; pseudopposite leaves below inflorescence absent	***A.schugnanica***
–	Subshrub growing in lowlands and foothills; leaves oblong; pseudopposite leaves below inflorescence present in flowering and early fruiting	***A.pseudotatarica* sp. nov.**
5	Plant with tumble-weed habit; stem erect with spreading branches; leaves rhombic or ovate; bract-like cover of female flowers either smooth or with thorn-like outgrowths (on the same plant)	***A.centralasiatica***
–	Plant not forming tumble-weed habit; leaves oblong; bract-like cover smooth or with 1–2 small outgrowths	***A.pamirica***

### ﻿List of native *Atriplex* species in India

**1. *Atriplexcentralasiatica* Iljin, Act. Inst. Bot. Ac. Sci. USSR, ser. 1, 2: 124 (1936).**

**Holotype.** [KAZAKHSTAN] Lac. Balchasch, prope Aczie [Balkhash Lake, near Aqshi], 19 Sep 1930, *E. Czerniakowska 819* (LE!).

This species was reported from India for the first time by Sukhorukov et al. (2019) and is distributed in Jammu and Kashmir State.

**2. *Atriplexpamirica* Iljin, Acta Inst. Bot. Ac. Sc. USSR, ser. 1, 2: 124 (1936).**

≡ Atriplextataricavar.pamirica (Iljin) G.L.Chu in Kung & Tsien, Fl. Reipubl. Pop. Sin. 25(2): 46 (1979), nom. inval. (Art. 41.5).

**Holotype.** [TAJIKISTAN] Khargosh, in ripa lac. Kara-kul [bank of Kara-kul Lake], 30 Jul 1878, *Yu. Ashurbaev s.n.* (LE!).

This species is also restricted to Jammu and Kashmir State (Sukhorukov et al. 2019). A varietal rank of this taxon cannot be accepted, because *A.tatarica* and *A.pamirica* occupy distant positions on the molecular tree (Žerdoner Čalasan et al. 2022) and belong to different sections, A.sect.Obionopsis (Lange) Dumort. and A.sect.Obione (Gaertn.) Reichenb., respectively (Sukhorukov et al. 2022).

**3. *Atriplexpseudotatarica* Sukhor. & Nidhan Singh (this paper).**

**4. *Atriplexrepens* Roth, Nov. Pl. Sp.: 377 (1821).**

≡ *Obionerepens* (Roth) G.L. Chu, Gen. New Evol. Syst. World Chenopod.: 165 (2017). Neotype (designated by Turner (2021: 373)): INDIA. *J.P. Rottler s.n.* (K barcode K001129778!, excluding material marked with a pencil cross; isoneotype G-DC barcode G00687837).

= *Obionenummularia* Moq., Chenop. Monogr. Enum.: 72 (1840).

≡ *Obionekoenigii* Moq. in DC., Prodr. 13(2): 109 (1849), nom. illeg. superfl. Holotype: INDIA. “Ex India orientali”, *J.P. Rottler s.n.* (G-DC barcode G00687837, isotype K barcode K001129778!).

– *Atriplexkoenigii* Wall., Numer. List: no. 6951 (1832), nom. nud.

– *Atriplexrepens* B.Heyne in herb.

**Note.** The name *Obionenummularia* Moq. was validly published on the basis of the only specimen (holotype) originating from India, *J.P. Rottler s.n.* collected in the late 18^th^ century and received by A. de Candolle under the name “*Atriplexcristata* Koenig” from M.N. Puerari (now at G-DC).

Zhu et al. (2003) and Zhu and Sanderson (2017) reported the presence of *A.repens* on Hainan Island (southern China); however, the latter species is distributed in the coastal areas in southern India (Karnataka, Kerala, Tamil Nadu, and Andhra Pradesh) and Sri Lanka. The correct name for the plants growing in Hainan and other parts of tropical China as well as in southern Japan is *A.maximowicziana* Makino.

**5. *Atriplexschugnanica* Iljin, Acta Inst. Bot. Acad. Sc. URSS, ser. 1, 2: 123 (1936).**

= *Chenopodiumpallidum* Moq., Chenop. Monogr. Enum.: 30 (1840), nom. rejic. prop.

≡ *Atriplexpallida* (Moq.) Sukhor., Phytotaxa 226(3): 288 (2015). Lectotype (designated by Sukhorukov and Kushunina (2014: 14)): [Probably NE INDIA] Voyage de V. Jacquemont aux Indes Orient., *Jacquemont 1377* (P barcode P04993339!, isolectotypes P barcodes P00606416! P04993338! P05047853!). Image of the lectotype available at: https://science.mnhn.fr/institution/mnhn/collection/p/item/p04993339

**Lectotype.** (designated by Sukhorukov and Tscherneva in Sukhorukov (2006: 384)): [TAJIKISTAN] Roschan [Roshan], Usoj [Usoy], in ripa flum. Bartanga [bank of Bartanga River], in decliviis lapidosis [rocky slopes], 20 Aug 1897, *S. Korshinsky 4692* (LE!, isolectotype LE!).

**Note.** The name *Chenopodiumpallidum* Moq. was proposed for rejection by Mosyakin and Mandak (2021) due to nomenclatural collisions with its typification (Sennikov 2022). Present in North Himalaya to Pamir Mountains: North India (Jammu & Kashmir, Himachal Pradesh, Uttarakhand), North Pakistan, Afghanistan, Tajikistan (Sukhorukov et al. 2019).

**6. *Atriplexstocksii* Boiss., Diagn. Pl. Orient., ser. 2, 4: 73 (1859).**

≡ Atriplexgriffithiivar.stocksii (Boiss.) Boiss., Fl. Orient. 4: 916 (1879).

≡ Atriplexgriffithiisubsp.stocksii (Boiss.) Boulos, Nordic J. Bot. 11(3): 310 (1991). Lectotype (designated by Hedge (1997: 84)): [PAKISTAN] Scinde [Sindh prov.], *J.E. Stocks 452* (G-Boiss, isolectotypes K barcode K000898566!, K000898567!).

= *Obionestocksii* Wight, Icon. Pl. Ind. Orient. 5(2): 5, tab. 1789 (1852). Lectotype (designated here): [PAKISTAN] Scinde [Sindh prov.], *J.E. Stocks 452* (K barcode K000898566!, isolectotypes K barcode K000898567!, G-Boiss).

**Notes.** The species was originally described as *Obionestocksii* Wight based on a single specimen, *J.E. Stocks 452*, collected in present-day Sindh Province of Pakistan. Boissier (1859) re-described the species as *Atriplexstocksii* based on two collections by Stocks from Pakistan and one from southern Iran, *Aucher-Eloy 5268*. In the protologue of *A.stocksii*, Boissier cited the type collection used by Wight but employed the same species epithet. For this reason, the protologue of his species name included the type of Wight’s species but Boissier’s species name cannot be treated as illegitimate. Boissier explicitly described his species as new and validly published its name in its own right, without any presumed basionym; for this reason, this species name has no basionym even though the potential basionym exists. As Boissier’s species name is not superfluous, it cannot be automatically typified by the type of Wight’s species name.

Wight (1852) used a single specimen to describe his new species, now stored at K, which is, however, not the holotype due to the availability of another element, i.e. an illustration published in the protologue. Boissier (1859) used three specimens, hence lectotypification is also needed. Hedge (1997: 84) indicated that the type of *A.stocksii* Boiss. is the specimen kept at G-DC, which belongs to the same gathering as the type of *O.stocksii* Wight. The later type designation with the specimen at K, which was made by Omer (2001), has no standing.

The species is reported from Gujarat and Tamil Nadu States (Rao 1986; Paul 2012), but in the latter state its presence is dubious. Also present in Rajasthan State.

### ﻿List of *Atriplex* species previously reported from India and hereby excluded from this country

1. *Atriplexcrassifolia* C.A.Mey. Occurs only in Kazakhstan, Russia (South-West Siberia), and western China (Xinjang). Reported by many old and recent authors (see above).

2. *Atriplexlaciniata* L. European coastal plant. Previously reported by Aitchinson (1869).

3. *Atriplexsagittata* Borkh. (= *A.nitens* Schkuhr). Species native to temperate regions of Eurasia. Previously reported by Paul (2012, as *A.nitens*).

4. *Atriplexrosea* L. Reported by Hooker (1890) and Paul (2012). Native to the Mediterranean, and Asia Minor, with recent scattered records in the Black Sea region and other parts of Europe (Sukhorukov 2006; Sukhorukov et al. 2022).

## Supplementary Material

XML Treatment for Atriplex pseudotatarica

## ﻿Additional information

### Author ORCIDs

Alexander P. Sukhorukov https://orcid.org/0000-0003-2220-826X

Nidhan Singh https://orcid.org/0000-0002-1155-1807

Maria Kushunina https://orcid.org/0000-0003-2541-6494

Maxim A. Zaika https://orcid.org/0000-0003-2633-4730

Alexander N. Sennikov https://orcid.org/0000-0001-6664-7657

### Data availability

All of the data that support the findings of this study are available in the main text or Supplementary Information.
